# Which Exercise Protocol Is Optimal for Improving Lower Crossed Syndrome in Athletes? Core Stability or NASM

**DOI:** 10.1002/hsr2.72471

**Published:** 2026-05-03

**Authors:** Milad Hassani, Seyed Hossein Hosseinimehr

**Affiliations:** ^1^ Department of Physical Education and Sport Sciences, Faculty of Humanities and Social Sciences University of Kurdistan Sanandaj Kurdistan Iran

**Keywords:** core stability exercises, Lower crossed syndrome, lumbar lordosis angle, McGill tests, NASM exercises, pelvic tilt

## Abstract

**Background and Aim:**

Lower crossed syndrome (LCS) is a common postural dysfunction among individuals with sedentary lifestyles; however, it can also affect athletes due to sport‐specific demands. This study aimed to investigate and compare the effects of core stability exercises (CSE) and National Academy of Sports Medicine (NASM) exercises on lumbar lordosis, pelvic tilt, and trunk muscle endurance in athletes with LCS.

**Methods:**

This study employed an experimental design with pretest and posttest assessments across three groups. Thirty‐six male athletes with LCS (aged 20–30 years) were randomly assigned to three groups: the CSE group, the NASM group, and the control group. Participants in the experimental groups performed their respective exercise protocols for 8 weeks (3 sessions per week). Lumbar lordosis and pelvic tilt angles were assessed using a flexicurve and a pelvic inclinometer, respectively. Additionally, the McGill test was used to determine the endurance of the trunk muscles.

**Results:**

Both exercise protocols significantly decreased the lumbar lordosis angle and pelvic tilt, and significantly increased the endurance of the trunk flexors and extensors (*p* < 0.05). However, the NASM protocol significantly increased the lateral trunk flexors on both the dominant side (*p* < 0.001) and the nondominant side (*p* = 0.005). Additionally, the findings revealed that the effects of 8 weeks of exercise on the lordosis angle (*F*(2, 33) = 0.153, *p* = 0.85, *ηp*² = 0.009), pelvic tilt (*F*(2, 33) = 2.01, *p* = 0.15, *ηp*² = 0.109), trunk flexor endurance (*F*(2, 33) = 2.875, *p* = 0.07, *ηp*² = 0.148) and extensor endurance (*F*(2, 33) = 1.246, *p* = 0.301, *ηp*² = 0.07) did not differ significant between the NASM and CSE group (*p* > 0.05); However, a significant difference was observed in the endurance of trunk lateral flexors on the dominant (*F*(2, 33) = 16.897, *p* < 0.001, *ηp*² = 0.506) and nondominant sides (*F*(2, 33) = 16.931, *p* < 0.001, *ηp*² = 0.506) among the groups and post hoc Tukey tests revealed that the NASM group exhibited significantly greater improvements than the CSE group (*p* < 0.001).

**Conclusions:**

An 8‐week of core stability and NASM exercise program are recommended to reduce the lumbar lordosis angle and pelvic tilt and to improve the endurance of trunk flexors and extensors in athletes with LCS. Although only the NASM protocol increased the endurance of lateral trunk flexors on both the dominant and nondominant sides, no significant difference was observed between the two exercise protocols regarding the other variables.

## 1 Background

Lower crossed syndrome (LCS), also known as distal crossed syndrome or pelvic crossed syndrome, is characterized by shortening or tightness of the lumbar erector spinae, iliacus, and rectus femoris muscles [1, 2, 3]. Additionally, this syndrome involves weakness and lengthening of the deep abdominal muscles anteriorly, as well as weakness and lengthening of the gluteus maximus and medius muscles posteriorly [1, 2, 3]. This muscle imbalance can lead to joint dysfunction, particularly at the L4–L5 and S1 vertebrae, the sacroiliac joints, and the hip joints [2, 3]. Common postural alterations include anterior pelvic tilt, increased lumbar lordosis, foot external rotation, and knee hyperextension. Pelvic muscle imbalances are more prominent when lumbar lordosis is pronounced and localized, whereas trunk muscle imbalances predominate when lordosis is milder and extends into the upper back [3]. Furthermore, simultaneous contraction of the abdominal muscles and hip extensors produces a posterior pelvic tilt, whereas concurrent activation of the hip flexors and lumbar extensors results in an anterior pelvic tilt. Altered pelvic tilt and lumbar curvature disrupt the length–tension relationship, placing certain muscles in a lengthened state and others in a shortened position [4, 5]. Previous studies have shown that athletes are more prone to postural deviations than non‐athletes due to sport‐specific demands [6]. LCS is more prevalent in sports involving repetitive movements and muscular imbalances in the hip and back, such as equestrian sports, cycling, and martial arts. Nevertheless, any athlete with lumbopelvic muscular imbalances may be susceptible to LCS [1]. Optimal posture is essential for athletic performance across most sports. The degree of lumbar lordosis is one of the primary indicators of postural disorders [7]. Excessive lumbar lordosis represents a postural deviation that compromises the body's natural alignment [2, 7]. Because the spine functions as a kinetic chain, alterations in one segment can produce compensatory changes in adjacent regions [8, 9]. Compared with neutral spinal alignment, lordotic postures increase compressive loading on the posterior vertebral elements, thereby placing greater stress on the intervertebral discs [8, 9]. Core musculature, including the abdominals, multifidus, pelvic floor, and diaphragm, plays a crucial role in maintaining lumbopelvic stability and mitigating spinal deviations. When appropriately strengthened, these muscles enhance neuromuscular control and support optimal spinal alignment, particularly within the lumbopelvic region [4]. Core stability exercises (CSEs) are designed to optimize core muscle activation, thereby facilitating efficient force generation and transfer from the spine to the lower extremities. Furthermore, CSEs are highly effective interventions for enhancing sports performance and improving rehabilitation outcomes [10]. Numerous studies have demonstrated that CSEs improve athletic performance, stability, and strength while reducing the risk of musculoskeletal injuries [10, 11, 12, 13]. The National Academy of Sports Medicine (NASM) Corrective Exercise Continuum is another widely used approach, comprising four phases: inhibit (release), lengthen (stretch), activate (strengthen), and integrate [14]. In the inhibit phase, tight or shortened soft tissues are addressed through self‐myofascial release; this is followed by the lengthen phase, which targets the same restricted musculature. Specifically, the inhibit phase aims to alleviate chronic myofascial trigger points that contribute to muscle stiffness and shortening. The lengthen phase restores optimal resting muscle length and improves the length–tension relationship. Finally, the activate and integrate phases emphasize functional movements that not only strengthen target muscles but also enhance neuromuscular coordination [14].

## 2 Methods

### 2.1 Study Design

This experimental study employed a pretest–posttest design with three groups: the CSE exercise group, the NASM training group, and a control group. Following participant selection and random allocation, baseline measurements were obtained for the lumbar lordosis and pelvic tilt angles (degrees) and for trunk muscle endurance (including flexors, extensors, and dominant and non‐dominant lateral flexors; measured in seconds). The experimental groups then completed an 8‐week training program based on the CSE and NASM protocols, while participants in the control group continued their usual daily and sport‐specific activities without additional intervention. After the training period, posttest measurements were conducted to assess the effects of the interventions. Ethical approval was granted by the Research Ethics Committee of the University of Kurdistan (IR.UOK.REC.1404.007), and all procedures complied with the Declaration of Helsinki. Written informed consent was obtained from all participants prior to their participation. Trial Registration was OSF, August 15th, 2025, https://doi.org/10.17605/OSF.IO/M726H.

### 2.2 Participants

A total of 36 male collegiate athletes were recruited and randomly assigned to one of three groups: the CSE group (*n* = 12), the NASM exercise group (*n* = 12), and the control group (*n *= 12). Participants represented various sports disciplines, including taekwondo (*n* = 12, 33.3%), football (*n* = 9, 25%), volleyball (*n* = 9, 25%), and kung fu (*n* = 6, 16.7%), and were evenly distributed across groups. Initially, 45 eligible athletes enrolled (15 per group); however, nine participants were excluded due to excessive absences (defined as missing more than two consecutive or three nonconsecutive sessions). Descriptive characteristics of the final sample are presented in Table 1. Sample size adequacy was evaluated via a post hoc power analysis using G*Power 3.1. Using *α* = 0.05, an effect size of *f* = 0.40, and *N* = 36 (12 per group), the achieved power (1 − β) was 0.87, indicating sufficient statistical power to detect medium‐to‐large effects. Inclusion criteria were: (1) a lumbar lordosis angle > 47° [15, 16]; (2) a pelvic tilt > 7° [17, 18]; (3) absence of cardiorespiratory disorders; and (4) no history of spinal surgery. The exclusion criterion was missing more than two consecutive or three nonconsecutive training sessions.

**Table 1:** Profile of research participants.

Group	Age (year)	Height (cm)	Mass (kg)	Sport experience (year)
CSE (*n* = 12)	25.4 ± 4.6	174.9 ± 8.4	68.1 ± 8.4	5.2 ± 1.9
NASM (*n* = 12)	24.8 ± 4.2	172.8 ± 6.1	70 ± 8.1	5.1 ± 1.9
Control (*n* = 12)	24.5 ± 4.7	174.1 ± 8.1	70.4 ± 9.9	5.9 ± 1.6
*p*‐value	0.9	0.25	0.44	0.13

Abbreviations: CSE, core stability exercises; NASM, National Academy of Sports Medicine exercises.

### 2.3 Randomization

**Figure 1:**
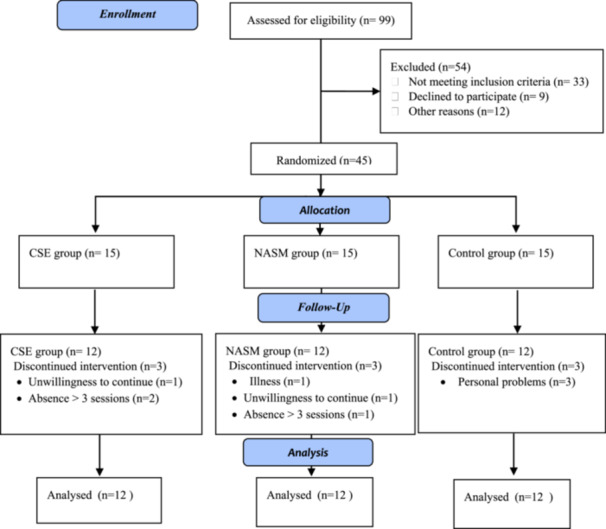
Flowchart of participant recruitment, allocation, and analysis.

After initial evaluations, participants were allocated to one of three groups: CSE, NASM, or a control group, using a sealed‐envelope method with equal allocation (1:1:1). An independent statistician, uninvolved in participant recruitment, intervention delivery, or data analysis, generated the random allocation sequence using a computer‐based random number generator with randomly permuted block sizes (3 and 6). This approach ensured balanced allocation over time and minimized selection bias. To maintain allocation concealment, the allocations were placed in sequentially numbered, opaque, sealed envelopes prepared by the statistician. Only the researcher responsible for enrollment and randomization had access to these envelopes, which were opened only after participants provided informed consent and completed all baseline assessments, thereby preserving concealment until randomization. Due to the nature of the CSE and NASM interventions, blinding of participants and instructors was not feasible. However, outcome assessors during follow‐up visits were blinded to group allocation. These assessors, who were not involved in recruitment, randomization, or intervention delivery, were explicitly instructed not to discuss group allocation with participants. Data analysts also remained blinded during data processing and primary statistical analyses, with group codes unmasked only after these analyses were finalized. Since baseline assessments were conducted prior to randomization, assessors were inherently blinded to group allocation. The participant flow diagram is presented in Figure 1.

After selecting the participants and dividing them into three groups, pre‐tests were taken to assess lumbar lordosis angle, pelvic tilt angle, and trunk muscle endurance. Then, core stability and NASM exercises were carried out for 8 weeks, and after completing the exercise protocols, post‐tests were taken. The control group performed daily and sports activities related to their sports disciplines during the training period, and only the experimental groups (CSE & NASM) performed training interventions other than daily and sports activities specific to their disciplines.

### 2.4 Baseline Measurements

All measurements were conducted by an experienced sports medicine and corrective exercise specialist (MH) at the University of Kurdistan laboratory. The examiner was blinded to the participants' group assignments. To measure lumbar lordosis, a flexicurve (flexible ruler) (CEB Flexible curve ruler, 30 cm, China) was used. Previous studies have reported that the flexicurve has high to very high reliability and moderate to high validity for measuring lumbar lordosis [19]. The degree of pelvic tilt was measured using a pelvic inclinometer (B0CHND2NPB, Texon Corporation, China). Previous studies have reported that the inclinometer exhibits good interrater reliability and test‐retest reliability within a single testing session, whereas test‐retest reliability between sessions is moderate‐to‐good [20]. Additionally, trunk muscle endurance was assessed using the McGill test. Lua et al. (2005) reported that the McGill test is reliable and valid for the assessment of trunk muscle endurance [21]. The participants' body mass and height were measured using a digital scale (Exacta, Nassau, Germany) and a tape measure. The reliability of the instruments was confirmed through a pilot study with 15 participants, focusing on consistency for a single evaluator. Intra‐rater reliability testing was conducted, and the intraclass correlation coefficients (ICC(3,1)) were calculated using a two‐way mixed‐effects model for absolute agreement based on single measurements. The ICC values ranged from 0.89 to 0.96, indicating strong reliability, with 95% confidence intervals ranging from 0.74 to 0.97 and 0.91 to 0.99, depending on the specific outcome measures evaluated. This demonstrates that the instruments were highly consistent when used by the same rater across repeated testing sessions.

#### 2.4.1 Measurement of Lumbar Lordosis Angle

**Figure 2:**
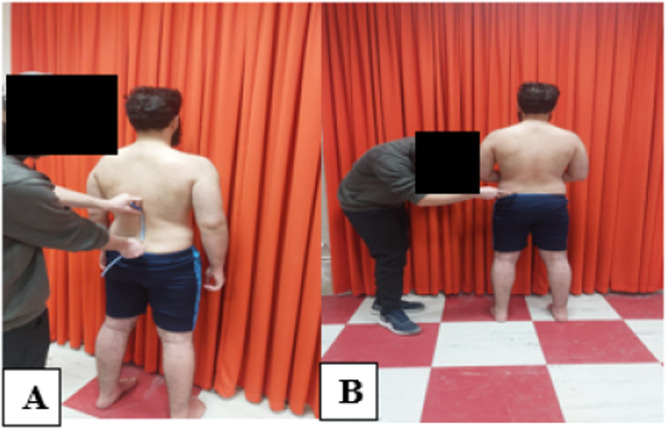
How to measure the lumbar curve (A) and pelvic tilt (B).

Given the flexicurve has many advantages, researchers have used it as a non‐invasive method for clinical evaluation and screening in healthy individuals, as well as for detecting spinal curvature abnormalities [19]. The flexicurve is a lightweight, inexpensive device that does not carry the repeated radiation risks associated with X‐rays [19]. To measure the lordosis angle, the participant should stand with their upper body exposed and feet positioned shoulder‐width apart. The participant then bends forward and places their hands on the table, and in this position, the examiner palpates the twelfth (or last) rib along the spine, thereby identifying the T12 vertebra. The identified landmark is then marked, and the participant is asked to stand upright. In the standing position, the examiner identifies the posterior superior iliac spines (PSISs) and marks the midpoint corresponding to the spinous process of the S2 vertebra. Next, the flexicurve is placed on the midline of the spine between the two previously determined points with full skin contact (Figure 2A), and after the arc conforms to the spinal contour, the device's outline is traced on graph paper without changing its shape. The origin and end of the L1 and S2 spinous processes, along with the deepest point of the lumbar curve, were marked on paper. The distance between these points is denoted by the symbol H, and the symbol L represents the depth of the arch. The lordosis angle was calculated using the formula *θ* = 4 arctan(2H/L) [19]. In this study, to enhance accuracy, each measurement was repeated twice for each participant, and the average of the two measurements was used in the final calculations.

#### 2.4.2 Measurement of Pelvic Tilt Angle

The measurement of pelvic tilt was performed with the participant in a comfortable standing position with their legs shoulder‐width apart [22]. The inclinometer was positioned so that one end was placed on the anterior superior iliac spine (ASIS) and the other end was placed on the posterior superior iliac spine (PSIS). In this position, the angle displayed on the inclinometer indicated the degree of pelvic tilt (Figure 2B) [20]. To improve accuracy, the angle was measured twice for each participant, and the average of the two measurements was used for analysis.

#### 2.4.3 Measurement of Trunk Muscle Endurance

**Figure 3:**
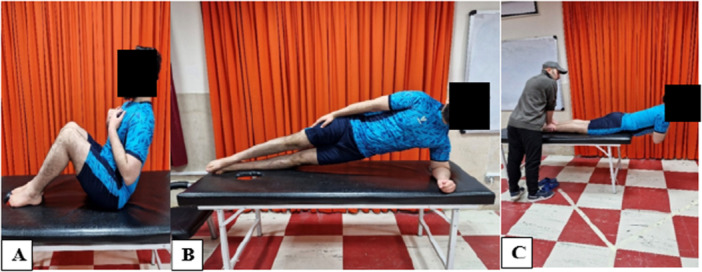
Flexor (A), lateral flexor (B), and trunk extensor (C) muscle endurance tests.

The McGill protocol was used to assess trunk muscle endurance (measured in seconds). It consisted of three separate tests. In the first test, which assessed trunk flexor endurance, the participant was positioned supine with the trunk at a 60° angle relative to the table (Figure 3A). In the second test, which evaluated the endurance of the lateral flexors, the participant lay on their side (tested separately for the dominant and nondominant sides). The shoulder was positioned at 90° of abduction, and the elbow was flexed at 90° and rested on the table (Figure 3B). The dominant side was defined as the side used for most daily and occupational activities, corresponding to the dominant hand (i.e., the hand used for throwing a ball). In the third test, which assessed the endurance of the trunk extensors, the participant lay prone on the examination table with the pelvis secured. In this position, the upper body from the ASIS upward was suspended off the table (Figure 3C) [23]. Participants were required to maintain each isometric position until volitional exhaustion. Each test was performed twice, with a 1‐min rest interval between trials [24]. The average time of the two valid trials was used for analysis [25].

#### 2.4.4 Core Stability Exercises (CSE)

The CSE protocol was conducted for 8 weeks (with 3 sessions per week). Each session lasted approximately 1 h, including 10 min of warm‐up, approximately 45 min of core exercises, and 5 min of cool‐down. The CSE comprised seven exercises: standard sit‐ups, sit‐ups with trunk rotation, reverse hyperextensions, V‐crunches, full planks, abdominal bridges, and side planks. Progressive overload was achieved by increasing training duration, decreasing rest intervals, increasing movement complexity, and reducing reliance on external support. Specifically, during the first and second weeks, participants performed 3 sets of 30 s with a rest‐to‐exercise ratio of 3:1; during the third and fourth weeks, 3 sets of 40 s with a ratio of 2:1; during the fifth and sixth weeks, 3 sets of 50 s with a ratio of 2:1; and during the seventh and eighth weeks, 3 sets of 60 s with a ratio of 1:1 (Table 2) [11].

**Table 2:** Core stability exercise protocol.

	Weeks 1 and 2	Weeks 3 and 4	Weeks 5 and 6	Weeks 7 and 8
Standard sit‐ups (C.E.C)	3 × 30 repetitions	3 × 40 repetitions	3 × 50 repetitions	3 × 60 repetitions
Sit‐ups with trunk rotation (C.E.C)	3 × 30 repetitions	3 × 40 repetitions	3 × 50 repetitions	3 × 60 repetitions
Reverse hyperextension (I.C)	Holding 3 × 30 s	Holding 3 × 40 s	Holding 3 × 50 s	Holding 3 × 60 s
V crunch (C.E.C)	Holding 3 × 30 s	Holding 3 × 40 s	Holding 3 × 50 s	Holding 3 × 60 s
Plank (I.C)	Holding 3 × 30 s	Holding 3 × 40 s	Holding 3 × 50 s	Holding 3 × 60 s
Abdominal bridge (I.C)	Holding 3 × 30 s	Holding 3 × 40 s	Holding 3 × 50 s	Holding 3 × 60 s
Side plank (I.C)	Holding 3 × 30 s	Holding 3 × 40 s	Holding 3 × 50 s	Holding 3 × 60 s
The ratio of exercise to rest	1:3	1:2	1:2	1:1

Abbreviations: C.E.C, concentric‐eccentric contraction; I.C, isometric contraction.

### 2.5 NASM Exercises

The NASM exercises, similar to CSE, were performed 3 days a week for 8 weeks. Each session lasted approximately 1 h, comprising 10 min of warm‐up, approximately 45 min of NASM exercises, and 5 min of cool‐down. The NASM exercise protocol included four technique phases: inhibition, stretching, activation, and integration (Table 3).

**Table 3:** NASM exercise protocol.

Week	Inhibition (Phase 1)—Foam Rolling (hard type)	Stretching (Phase 2)—Hold at first resistance	Activation (Phase 3)—10–15 reps, 1–2 s isometric at end ROM + 1 s eccentric	Integration (Phase 4)—Dynamic multi‐joint functional movements	Progression Notes (Overload)	Sets/Total Time per Phase
1–2	Calves, quadriceps, TFL/IT band, thoracic spine, latissimus dorsi (30 s per area, slow rolls + pause on tender spots)	Static hold: Hip flexor stretch, quadriceps stretch, hamstring stretch, pectoral stretch (30 s per side)	Glute bridge (10 reps), bird dog (10/side), clamshell (10–12/side), dead bug (10/side)	Bodyweight squat to overhead reach, multiplanar lunge, step‐up to balance, plank to push‐up	Baseline – focus on technique & mind‐muscle connection	1–2 sets per phase; ~8–10 min per phase
3–4	Same areas + add adductors & upper trapezius/levator scapulae (35–40 s per area, increase pressure)	Static + neuromuscular: Add contract‐relax to hip flexor & TFL stretch (35–40 s hold)	Glute bridge with 2 s hold at top (12 reps), side‐lying hip abduction (12/side), prone cobra (12 reps), cable pull‐through or band glute kickback (12 reps)	Single‐leg Romanian deadlift (bodyweight), lateral lunge to balance, push‐up with rotation, squat jump to stabilization	Increase reps + hold time; add light band resistance if form is perfect	2 sets per phase; ~10–12 min per phase
5–6	Same + focus slower rolls on key tight areas (40–50 s per area, add trigger point pause 10–15 s)	Increase to 45 s hold; add active‐assisted stretches (e.g., partner or strap for hamstring)	Glute bridge march (single‐leg alternation, 12–15 reps), monster walk with band (15 steps/side), quadruped hip extension (15/side), pallof press (12/side)	Lunge with overhead medicine ball reach, step‐up to knee drive, single‐leg squat to box, rotational cable chop	Add external load (light dumbbell/medicine ball 2–5 kg); increase reps & complexity	2–3 sets per phase; ~12 min per phase
7–8	45–60 s per area, emphasize deep pressure & full coverage (add percussion if available)	45–60 s hold; combine static + active stretch (e.g., PNF contract‐relax)	Single‐leg glute bridge (12–15/side with 2–3 s hold), banded hip abduction + external rotation (15 reps), Turkish get‐up prep (light), plank variations with leg lift	Multiplanar lunge matrix, single‐leg deadlift to row, medicine ball slam to overhead throw, dynamic plank to pike	Maximal progression: higher reps, added speed/load, advanced patterns; monitor fatigue	3 sets per phase; ~12–15 min per phase

Inhibition techniques (Phase 1): This technique was used to release tension and reduce the overactivity of neuromyofascial tissues. In this study, high‐density foam rolling was used to release overactive neuromyofascial tissues. Foam rolling increases pressure on soft tissue structures and accesses deep layers of fascia. In this phase, the participant was instructed to move the foam roller over the tight soft tissue for 30 s [14, 26].

Stretching techniques (Phase 2): These techniques were used to increase the elasticity, length, and range of motion of the neuromyofascial tissues. Each stretch was held at the first point of resistance for 30 s.

Activation techniques (Phase 3): This technique was used to retrain or increase the activity of underactive muscles (abdominals, gluteus maximus, gluteus medius, and gluteus minimus). These exercises were performed for 10 to 15 repetitions, with each repetition consisting of a 1‐ to 2‐second isometric contraction at the end of the range of motion, followed by a 1‐second eccentric contraction [14, 24].

Integration techniques (Phase 4): The exercises in this phase were used to retrain and coordinate neuromuscular function through progressive functional movements using dynamic exercises designed to enhance the coordination of stabilizing and prime mover muscles.

### 2.6 Statistical Analysis

SPSS statistical software (Version 26.0, SPSS Inc., Chicago, IL) was used for all statistical analyses. The Shapiro–Wilk test was used to assess the normality of the data distribution. After confirming that the data were normally distributed, a 2 × 3 mixed‐model ANOVA was conducted to examine within‐ and between‐group effects. Additional post‐hoc comparisons were performed using the Tukey test. Additionally, a paired‐samples *t*‐test was used to compare pretest and posttest results within each group, with the significance level set at 0.05.

## 3 Results

Based on the distribution‐based approach, the minimal clinically important difference (MCID) was estimated using the formulas MCID = 0.5 × SD and MCID = 1 × SEM, where SD represents the standard deviation and SEM represents the standard error of measurement. The calculated MCID values indicated that the pelvic tilt ranged between 0.52° and 1.05°, lumbar lordosis ranged between 1.57° and 2.53°, trunk flexor endurance ranged between 12 and 17 s, trunk extensor endurance ranged between 13 and 18 s, dominant‐side trunk lateral flexor endurance ranged between 8 and 11 s, and non‐dominant‐side trunk lateral flexor endurance ranged between 10 and 14 s. These thresholds suggest that changes exceeding these values can be interpreted as clinically meaningful improvements beyond normal measurement variability.

**Figure 4:**
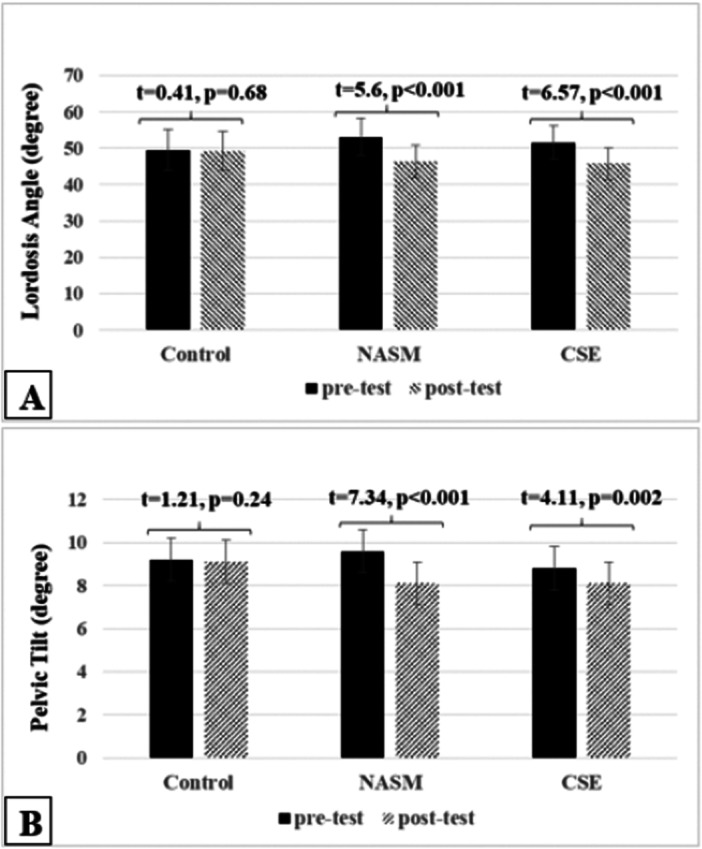
Comparison (mean ± SD) of the lordosis angle (A) and pelvic tilt (B) among the three groups. CSE, core stability exercises; NASM, National Academy of Sports Medicine exercises.

Table 4 and Figure 4 present the descriptive statistics for the research variables across the three groups and the results of the paired‐samples *t*‐test. Paired‐samples *t*‐tests indicated that the 8‐week NASM and CSE intervention led to significant reductions in lumbar lordosis angle (Figure 4A) and pelvic tilt (Figure 4B), as well as significant improvements in trunk flexor and extensor muscle endurance (Table 4) among athletes with LCS. However, only the NASM protocol showed a significant increase in the endurance of the trunk lateral flexor muscles on both the dominant and non‐dominant sides in athletes with LCS after 8 weeks. No significant changes were observed in the control group (Table 4).

**Table 4:** Pre‐ and post‐test of trunk muscle endurance in the three groups.

Variable	Core Stability Group (*n* = 12)				NASM Group (*n* = 12)		Control Group (*n* = 12)	
	Pre‐test (mean ± SD)	Post‐test (mean ± SD)			Pre‐test (mean ± SD)	Post‐test (mean ± SD)	Pre‐test (mean ± SD)	Post‐test (mean ± SD)
Flexors (s)	108.1 ± 30.2	118.1 ± 36.2			112.1 ± 48.2	122.1 ± 54.3	108.1 ± 30.1	108.1 ± 36.1
	df = 11, *t* = −4.8, *p *< 0.000, 95% CI: −40.8 to −15.42, effect size = 0.3				df = 11, *t* = −4.6, *p *< 0.000, 95% CI: −33.4 to −15.37, effect size = 0.19		df = 11, *t* = −1.06, *p* = 0.31, 95% CI: −10.1 to 3.52, effect size = 0.01	
Extensors (s)	150.2 ± 36.9	156.1 ± 36.2			162.2 ± 36.5	186.1 ± 43.2	150.2 ± 36.2	156.2 ± 31.3
	df = 11, *t* = −2.42, *p* = 0.034, 95% CI: −34.7 to −1.6, effect size = 0.16				df = 11, *t* = −4, *p *< 0.000, 95% CI: −36.3 to −12.3, effect size = 0.59		df = 11, *t* = −1.4, *p* = 0.18, 95% CI: −28.9 to 6.44, effect size = 0.17	
Dominant Lateral Flexors (s)	102.2 ± 24.6	109.1 ± 20.3			104.3 ± 48.2	148.1 ± 36.2	102.2 ± 30.6	114.1 ± 30.3
	df = 11, *t* = −3.11, *p* = 0.1, 95% CI: −19.9 to 20.3, effect size = 0.307				df = 11, *t *= −6.2, *p *< 0.000, 95% CI: −121.7 to −85.6 effect size = 1.03		df = 11, *t* = −1.33, *p* = 0.2, 95% CI: −30.1 to 15.17, effect size = 0.39	
Non‐dominant Lateral Flexors (s)	120.4 ± 30.5	119.4 ± 24.5			130.3 ± 45.3	178.1 ± 33.1	120.4 ± 30.5	108.4 ± 24.5
	df = 11, *t* = −2.46, *p* = 0.31, 95% CI: −39.1 to 3.7, effect size = 0.03				df = 11, *t* = −3.59, *p* = 0.005, 95% CI: −123.5 to −50.3, effect size = 1.21		df = 11, *t* = 0.911, *p* = 0.38, 95% CI: −28.7 to 23.7, effect size = 0.43	

Abbreviations: CSE, core stability exercises; NASM, National Academy of Sports Medicine exercises.

On the other hand, results of mixed‐model ANOVA (2 × 3) showed that the time and the interaction of time ×group had a significant effect on all of the dependent variables and the only interaction effect of time × group on trunk extensor muscle endurance was not significant (Table 5). Additionally, the findings revealed that the effects of 8 weeks of exercise on the lordosis angle (*F*(2, 33) = 0.153, *p* = 0.85, *ηp*² = 0.009), pelvic tilt (*F*(2, 33) = 2.01, *p* = 0.15, *ηp*² = 0.109), trunk flexor (*F*(2, 33) = 2.875, *p* = 0.07, *ηp*² = 0.148) and extensor endurance (*F*(2, 33) = 1.246, *p* = 0.301, *ηp*² = 0.07) were not significant between NASM and CSE group (*p *> 0.05); however, there was a significant difference in the endurance of trunk lateral flexors on the dominant (*F*(2, 33) = 16.897, *p* < 0.001, *ηp*² = 0.506) and nondominant sides (*F*(2, 33) = 16.931, *p* < 0.001, *ηp*² = 0.506) among the groups, and results of Tukey test shaowed the NASM group exhibited greater improvement than did the core stability (*p* < 0.001).

**Table 5:** Results of mixed‐model ANOVA test.

Variable	Factor	df	*F*	*p*‐value	*ηp*²
Lordosis angle	time	1	63.324	< 0.001	0.657
	time × group	2	14.25	< 0.001	0.463
Pelvic tilt	time	1	67.237	< 0.001	0.671
	time × group	2	18.045	< 0.001	0.522
Trunk flexor endurance	time	1	52.198	< 0.001	0.613
	time × group	2	9.033	0.001	0.354
Trunk extensor endurance	time	1	19.19	< 0.001	0.368
	time × group	2	0.85	0.436	0.49
Trunk dominant lateral flexor	time	1	24.088	< 0.001	0.422
	time × group	2	19.138	< 0.001	0.537
Trunk non‐dominant lateral flexor	time	1	22.332	< 0.001	0.404
	time × group	2	11.814	< 0.001	0.417

## 4 Discussion

The present study aimed to investigate and compare the effects of 8 weeks of NASM and CSE on the lumbar lordosis angle, pelvic tilt, and trunk muscle endurance in athletes with LCS. Statistical analysis showed that 8 weeks of core stability and NASM exercises significantly reduced the lumbar lordosis angle and pelvic tilt, and increased the endurance of trunk flexors and extensors in athletes with LCS. The statistical results of the study also revealed that only NASM exercises had a significant effect on the endurance of the lateral flexor muscles on both the dominant and nondominant sides. In addition, the effects of 8 weeks of exercise on the lordosis angle, pelvic tilt, trunk flexor and extensor endurance were not significant between the groups; however, there was a significant difference in the endurance of the trunk lateral flexors on the dominant and nondominant sides, with the NASM group demonstrating greater improvement than the core stability and control groups. No significant difference was found between the effects of these two types of exercise protocols on other variables (trunk flexor and extensor endurance). On the other hand, MCID analysis showed that the difference between pre‐test and post‐test values for various variables in the CSE group, although statistically significant, did not reach the MCID threshold. In contrast, when comparing pre‐test and post‐test results for the NASM group, except for the trunk flexor endurance variable, all other variables met the MCID threshold.

Lumbar lordosis is a crucial curvature of the spine, playing a vital role due to its strategic location and its connection to the pelvis. Maintaining a healthy spine is essential for optimal posture and overall physical well‐being [16]. This curvature is supported not only by bony structures but also by ligaments, muscles, and intervertebral discs. In the absence of adequate muscular engagement, the pelvic girdle cannot maintain optimal stability. Spinal core stability is supported by specific muscles such as the multifidus, transversus abdominis, and other deep trunk muscles. In individuals with LCS, these muscles often exhibit delayed activation, contributing to excessive lumbar curvature [27]. Segmental vertebral stability is maintained by these muscles, which provide targeted stabilization to specific spinal segments. If the strength and endurance of any muscle in the lumbar‐pelvic region decline, this can lead to altered pelvic alignment, disrupting postural balance and increasing susceptibility to musculoskeletal issues [16].

In LCS, the hip flexor muscles (e.g., iliacus and psoas) and lumbar extensors become short and tight, whereas the abdominal muscles, such as the rectus abdominis, and the hip extensor muscles, such as the hamstrings and gluteal muscles, become weak and lengthened. This muscle imbalance alters the static and dynamic posture of the body [2, 3, 28]. When this misalignment occurs, the body continuously attempts to compensate to maintain function; however, these adaptations ultimately exacerbate imbalance and functional impairment. Consequently, individuals with this condition frequently experience chronic back pain, piriformis syndrome, or anterior knee pain [9]. Since all parts of the body are connected through the kinetic chain of muscles and fascia, performing CSE appears to enhance proprioception in the core region [11, 29]. The muscles located in the core of the body, such as the abdominal muscles, multifidus, pelvic floor muscles, and diaphragm, play a major role in maintaining stability in the lumbopelvic region and mitigating spinal deviations. By stabilizing the spine and pelvis, these muscles facilitate both fundamental and complex functional movements. When effectively strengthened, these muscles can enhance neuromuscular control and support the optimal curvature of the spine, especially in the lumbopelvic region [3]. As previously mentioned, an increase in the lumbar lordosis angle and subsequent LCS occurs due to muscle imbalance in the lumbopelvic region, specifically because the shortened, tight lumbar extensors and hip flexors create a dominant force couple that overrides the weaker, lengthened hip extensors and abdominal muscles. As a result of this muscular imbalance, the anterior pelvic tilt and exaggerated lumbar lordosis increase, which compromises the stability of the lumbopelvic region. Therefore, to correct this condition, the shortened muscles must be stretched, and the lengthened, weakened muscles must be strengthened. By targeting and strengthening the lengthened abdominal musculature, CSE restores lumbopelvic muscular balance and subsequently reduces the lumbar lordosis angle [30, 31, 32].

On the other hand, as previously mentioned, NASM exercises use four stages to correct abnormalities. This approach, in addition to strengthening the lengthened muscles (common in) core stability exercises, also stretches the shortened muscles, thereby correcting the resulting muscle imbalance and allowing the muscles in this area to return to their normal state [14, 33]. It appears that 8 weeks of NASM exercises, through strengthening weak muscles, reducing tension in shortened muscles, improving movement patterns, and enhancing movement coordination, exerts positive effects on lumbar lordosis, pelvic tilt, and trunk muscle endurance. Since one of the primary objectives of this study was investigating the impact of exercise protocols (designed to address LCS) on lumbar lordosis and pelvic tilt angles in the experimental groups, the results revealed a significant improvement in both the core stability and NASM exercise groups after 8 weeks. The positive impact of these protocols suggests that, although the participants were college athletes, these exercises improved lordosis and pelvic tilt angles, even in the context of their sport‐specific demands. In contrast, no significant changes were observed in the lordosis and pelvic tilt angles of athletes who had LCS and continued only their sports training (control group). This highlights the negative consequences of omitting corrective training and underscores the importance of incorporating CSE and NASM exercises into the training routines of athletes with LCS. Furthermore, considering that the endurance of the core muscles in the exercise groups was significantly improved after 8 weeks, both CSE and NASM protocols effectively enhanced the endurance of these muscles. However, in the CSE group, this did not lead to a significant improvement in the lateral flexor muscles of the trunk. It appears that NASM exercises, owing to their greater variety, specificity, and attention to specific muscle groups, as well as the variety of muscle contractions (isometric, concentric, and eccentric) compared to CSE (which primarily utilizes isometric contraction only), can have a significant impact on improving the endurance of the trunk lateral flexor muscles in athletes with LCS. This finding requires further investigation, and it is recommended that future researchers explore it in greater depth.

## 5 Limitations

This study also has several limitations that should be acknowledged. First, although the study ensured that participants from various sports were evenly distributed across research groups, the total number of participants per sport varied. Second, the participants were exclusively male; therefore, the findings cannot be generalized to female athletes or the broader population. Third, although efforts were made to match training volume across both protocols (considering exercise type, number of sets, and rest intervals), practical constraints made complete adherence challenging. This limitation should be taken into account. Fourth, the participants were collegiate athletes in taekwondo, volleyball, football, and kung fu with 3–5 years of club experience; consequently, the results cannot be generalized to professional athletes, individuals with different training backgrounds, or participants in other sports.

## 6 Conclusion

Eight weeks of core stability and NASM exercises are recommended for athletes with LCS to help reduce the angle of lumbar lordosis and pelvic tilt, while simultaneously improving the endurance of the muscles responsible for flexing and extending the trunk. Notably, only the NASM exercises were effective in enhancing the endurance of lateral flexor muscles on both dominant and non‐dominant sides after this period.

## Author Contributions

**Milad Hassani:** writing – original draft, methodology, software, formal analysis, data curation, investigation, conceptualization, validation, visualization. **Seyed Hossein Hosseinimehr:** conceptualization, investigation, writing – original draft, writing – review and editing, visualization, validation, methodology, software, formal analysis, project administration, resources, supervision, data curation.

## Funding

The authors have nothing to report.

## Ethics Statement

All study protocols and materials were approved by the Research Ethics Committees of the University of Kurdistan (IR.UOK.REC.1404.007), and all procedures were performed following the Declaration of Helsinki. Trial Registration was OSF, August 15, 2025, https://doi.org/10.17605/OSF.IO/M726H (Retrospectively Registered).

## Consent

Informed consent was obtained from all the individual participants included in the study.

## Conflicts of Interest

The authors declare no conflicts of interest.

## Transparency Statement

The lead author Seyed Hossein Hosseinimehr affirms that this manuscript is an honest, accurate, and transparent account of the study being reported; that no important aspects of the study have been omitted; and that any discrepancies from the study as planned (and, if relevant, registered) have been explained.

## Data Availability

The data that support the findings of this study are available from the corresponding author upon reasonable request.
